# Incidental Synchronous ^99m^Tc-HYNIC-TOC Avid Lesion of the Neck in a Patient with Metastatic Melanoma: A Metastatic Lymph Node or a Carotid Body Tumor Masquerading As a Lymph Node?

**DOI:** 10.22038/AOJNMB.2021.59706.1415

**Published:** 2022

**Authors:** Vahid Roshanravan, Ehsan Soltani, Ehsan Hasanzadeh Haddad, Ramin Sadeghi, Azadeh Sahebkari, Mahdi Mottaghi, Atena Aghaee

**Affiliations:** 1 Nuclear Medicine Research Center, Mashhad University of Medical Sciences, Mashhad, Iran; 2 Surgical Oncology Research Center, Mashhad University of Medical Sciences, Mashhad, Iran; 3 Student Research Committee, Mashhad University of Medical Sciences, Mashhad, Iran

**Keywords:** ^99m^Tc-HYNIC-TOC, Melanoma, Carotid body tumor, Paraganglioma, ^99m^Tc-Phytate

## Abstract

A 53-year-old woman with a plantar malignant melanoma lesion was referred to our tertiary clinic for sentinel lymph node mapping. Lymphoscintigraphy with ^99m^Tc-Phytate detected ipsilateral inguinal and popliteal sentinel nodes. After total resection of nodes, the pathology report confirmed that all specimens were involved by the tumor. As part of an institutional study evaluating somatostatin receptor avidity of melanoma by ^99m^Tc-HYNIC-TOC scan, she also underwent a whole-body octreotide scan, which surprisingly showed intense tracer uptake in the right cervical region, confining in SPECT/CT images to a mass at the C2 spinal level, adjacent to the right carotid bifurcation. Neck surgery with gamma probe after injection of another dose of ^99m^Tc-HYNIC-TOC was performed successfully, and the pathology report was consistent with a carotid body tumor. To best our knowledge, our case is the first one in the literature, which reports an incidental paraganglioma with ^99m^Tc-HYNIC-TOC scan which resected via radio-guided surgery, again with ^99m^Tc-HYNIC-TOC tracer.

## Introduction

 Paragangliomas are rare, slow-growing, and usually benign extra-adrenal neuroendocrine neoplasms of chromaffin-cell origin. Para-ganglionic tissues in the carotid body region are called carotid paraganglioma (also known as chemodectoma or carotid body tumor) (1). They can arise from different areas and are named accordingly, such as glomus tympani-cum, glomus jugulare, vagal paraganglioma, pulmonary paraganglioma, etc. (1). About 15-35 % of symptomatic cases could be metastatic (2). MRI is the standard imaging modality, but whole-body imaging by ^99m^Tc-HYNIC-TOC is preferred for patients with risk factors for metastasis (i.e., larger tumors, high mitosis on 

pathologic study) (3). Best results are acquired using the Ga-68 DOTA-SSA; however, considering its low availability and high cost, a ^99m^Tc-HYNIC-TOC scan would be an appropriate alternative (4). We present a case of incidental paraganglioma in a patient with melanoma who underwent an experimental ^99m^Tc-HYNIC-TOC scan.

## Case Report

**Figure1:**
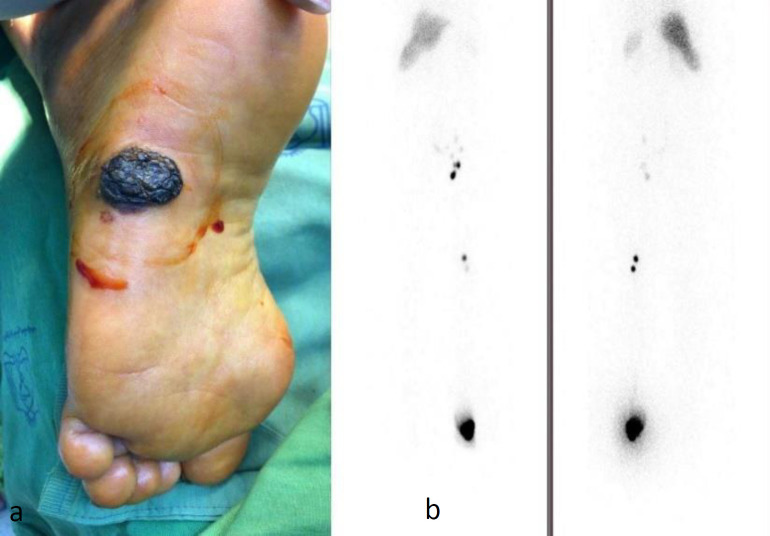
**a**. The primary lesion was located in the surface of the right foot. **b**. Lymphoscintigraphy images shows sentinel nodes in popliteal and inguinal regions

 A 53-year-old woman with a left plantar malignant melanoma lesion was referred to our department for sentinel lymph node mapping. Lymphoscintigraphy with ^99m^Tc-Phytate detected sentinel lymph nodes in popliteal and inguinal regions. After resection of all nodes, a pathologic study confirmed the tumoral involvement of all nodes (Figure 1).

 Then, we performed a ^99m^Tc-HYNIC-TOC scan to evaluate the tumoral somatostatin receptor avidity as a part of an approved institutional study (IR.MUMS.MEDICAL.REC. 1400.032).

**Figure2:**
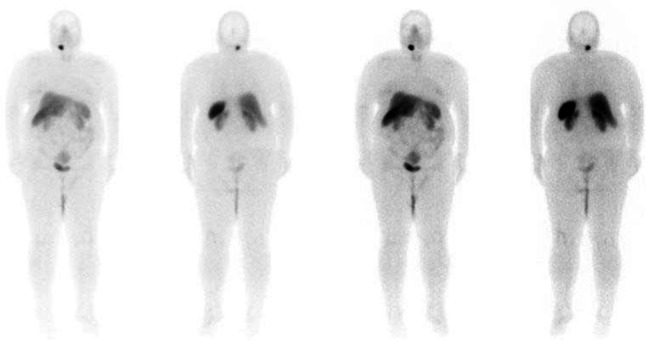
Whole body Tc99m-HYNIC- toc scan shows intense uptake in the right cervical region

 Anterior and posterior whole-body images were obtained with GE healthcare Discovery NM/CT 670 DR dual-headed gamma cameras. The protocol included a 30 to 40minute acquisition (10 cm/min bed reposition) on a 1024×256 matrix with the Low Energy-High Resolution collimators and application of 140 keV peaks with a 15% window, four hours after tracer injection. Whole-body views revealed physiologic biodistribution of radiotracer throughout the body and a focus of intense tracer accumulation in the right side of the neck. Further localization showed a mass in the C2 level on the SPECT/CT images. The lesion was located close to the right common artery bifurcation (Figure2).

**Figure3:**
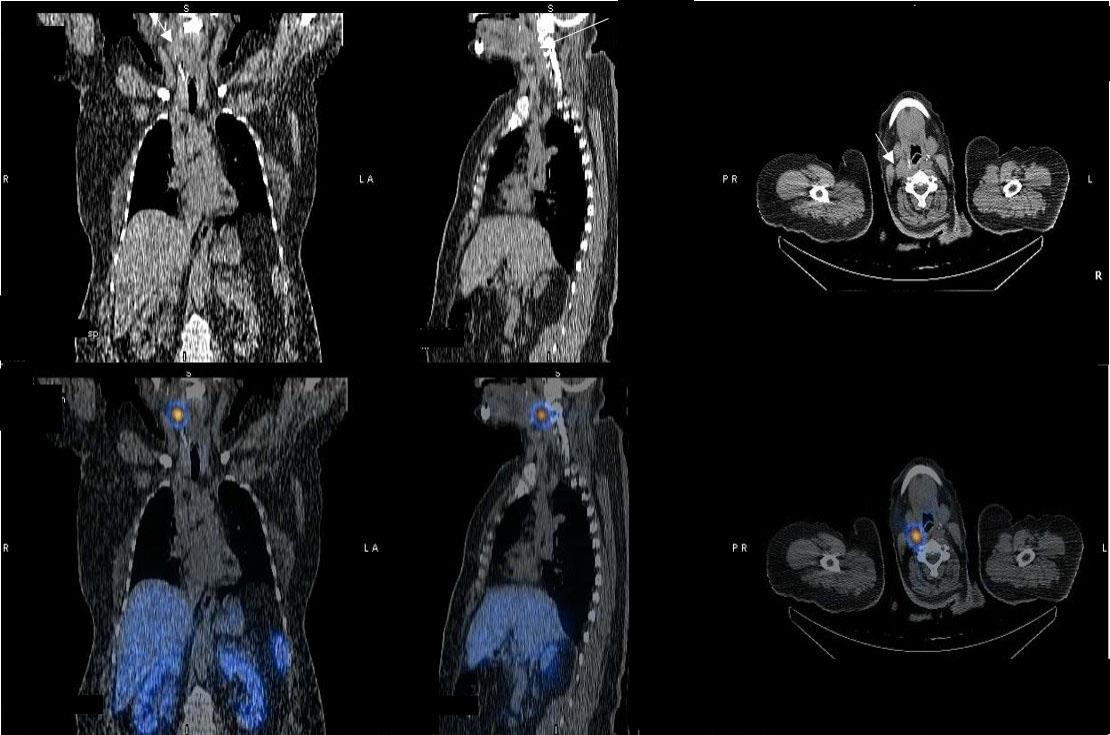
SPECT/CT images localized the Octero avid lesion to a mass in the right cervical level II

 SPECT/CT scan from the neck four hours after tracer injection revealed a 1.5 cm round hypodense lesion with soft tissue density. It was located adjacent to the right common carotid artery bifurcation and showed intense ^99m^Tc-HYNIC -TOC accumulation. These findings raised suspicion for a metastatic lymph node or a synchronous octreotide-avid neck tumor like a paraganglioma. The SPECT acquisition protocol includes a 30-minute stop and shot acquisition on a 64×64 matrix with the same collimators and peaks as mentioned previously. A Butterworth filter with that an order of 7.0 and a cutoff of 0.30 with attenuation and scatter correction were also used. A low dose CT scan was also performed simultaneously for attenuation correction and better localization of the mentioned lesion (Figure 3).

**Figure4:**
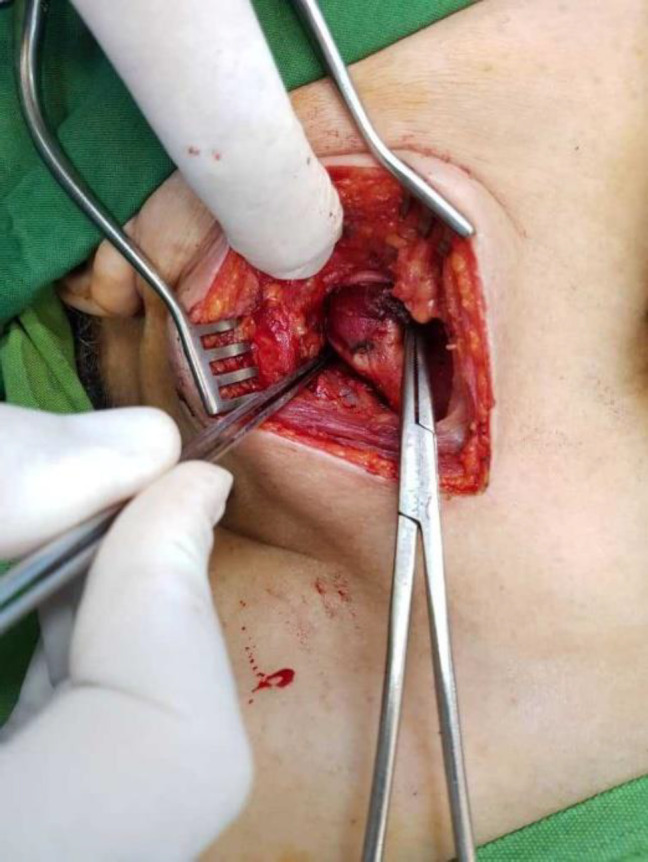
The mentioned octero avid mass was successfully found with gamma probe during surgery

 Since lymphatic metastasis to the neck is considered distant metastasis in plantar melanoma, the inguinal and popliteal complete dissections were postponed, and we decided to examine neck mass pathology first. The patient received 10 mCi of intravenous ^99m^Tc-HYNIC-TOC, two hours before the surgery. During the 

surgery, we used a gamma probe to precisely localize and remove the mass (Figure 4). Pathological studies confirmed the diagnosis of a carotid body tumor. The patient underwent a complete dissection of inguinal and popliteal nodes in the same week and was referred to the oncology clinic for follow-up**.**

## Discussion

We present an incidental paraganglioma in a patient suffering from melanoma who underwent an experimental ^99m^Tc-HYNIC -TOC scan. The importance of finding simultaneous melanoma and paraganglioma in a relatively middle- aged person raises suspicion for syndromic diseases; Wadt et al. proposed that mutations in BRCA1-associated protein-1 may make the patient susceptible to melanoma, para-ganglioma, and breast cancer. Diagnosis of these patterns, which are usually familial, is vital for 

future follow-ups of such patients (5). Additionally, even if we consider these situations sporadic, the accurate initial staging of melanoma is crucial to determine the prognosis and management (6). Multiple studies postulated different mechanisms for Peptide-targeted radionuclide therapy (7-10). Peptide-targeted therapeutic radionuclides, including ^177^Lu-DOTA-TATE, are developing methods that are used widely in the treatment of some somatostatin-expressing tumors, namely metastatic neuroendocrine tumors. Still, it warrants further investigation to be applied for metastatic octreotide-avid melanomas.

 In conclusion, our case is the first to describe an incidental finding of a paraganglioma by ^99m^Tc-HYNIC-TOC scan, which was also resected via radio-guided surgery after injection of this tracer. The ^99m^Tc-HYNIC-TOC scan could be a promising agent for detecting such lesions and associated metastases and radio-guided strategies to decrease surgical interventions and related morbidities.
